# Evolution of placentation in cattle and antelopes

**DOI:** 10.21451/1984-3143-AR2018-00145

**Published:** 2020-05-22

**Authors:** Anthony M. Carter

**Affiliations:** Cardiovascular and Renal Research, Institute of Molecular Medicine, University of Southern Denmark, Odense, Denmark.

**Keywords:** Antilopinae, binucleate trophoblast cell, Bovidae, Bovinae, chorioallantoic placenta, domestication, fetal membranes, placental hormones, placentome, unilateral implantation

## Abstract

Bovids have enjoyed great evolutionary success as evidenced by the large number of extant species. Several important domestic animals are from this family. They derive from both subfamilies: cattle and their kin belong to Bovinae and sheep and goats to Antilopinae. The premise of this review, therefore, is that evolution of reproduction and placentation is best understood in a context that includes antelope-like bovines and antelopes. Many key features of placentation, including hormone secretion, had evolved before bovids emerged as a distinct group. Variation nevertheless occurs. Most striking is the difference in fusion of the binucleate trophoblast cell with uterine epithelium that yields a transient trinucleate cell in bovines and many antelopes, but a more persistent syncytium in wildebeest, sheep and goat. There is considerable variation in placentome number and villus branching within the placentome. Many antelopes have right-sided implantation in a bicornuate uterus whilst others have a uterus duplex. Finally, there has been continued evolution of placental hormones with tandem duplication of *PAG* genes in cattle, differences in glycosylation of placental lactogen and the emergence of placental growth hormone in sheep and goats. The selection pressures driving this evolution are unknown though maternal-fetal competition for nutrients is an attractive hypothesis.

## Introduction

Cattle, buffaloes, sheep and goats account for around 80% of livestock (Bar-On, Phillips, and Milo, 2018) and are of great cultural and economic importance (Clutton-Brock, 2012). The domesticated species derive from a much greater pool of bovids that includes antelopes and antelope-like bovines. It is a premise of this review that the evolution of bovids, and of ruminants in general, is best understood by considering wild as well as domestic species. Using this approach, we shall consider how the reproductive strategy of bovids has contributed to their evolutionary success. 

The specific focus is placentation. The epitheliochorial placenta of ruminants is an advanced form of placentation that was derived from a more invasive one (Elliot and Crespi, 2009; Mess and Carter, 2006; Wildman *et al*., 2006; Carter and Mess, 2017). It is diffuse in the basal tragulids and cotyledonary in pecoran ruminants (Wooding and Burton, 2008). Placentation in Bovidae has not been reviewed since the work of Hradecký (Hradecky, 1986; Hradecky *et al*., 1988a, 1988b). There are variations in the number of uterine caruncles and fetal cotyledons as well as in the shape and internal structure of the placentomes. Binucleate trophoblast cells and their hormones are of especial interest and have undergone evolution at the ordinal and family level.

The narrative will begin with the remarkable ascent of the bovids and their later domestication. Next it will deal with various aspects of placentation, especially the fate of the binucleate trophoblast cell and the role of syncytin, the product of an endogenous retroviral gene, in its fusion with uterine epithelium to form a fetal-maternal hybrid cell or syncytium. To search for evolutionary trends, the account will proceed to variations in placentation among the twelve tribes of bovids as well as the placental hormones. A concluding section will refer to the ruminant and bovid trees and attempt to define the branching points at which innovations occurred. 

## Evolution and domestication of bovids

Relations between eutherian mammals have been clarified by molecular phylogenetics and phylogenomics. Four orders share a common ancestor: carnivores (Carnivora), horses and tapirs (Perissodactyla), pangolins (Pholidota) and even-toed ungulates (Cetartiodactyla). Three of these orders are characterized by epitheliochorial placentation whereas the endotheliochorial placentation of carnivores reflects an evolutionary reversal (Carter and Mess, 2017). Whales and dolphins are now counted among the even-toed ungulates (Spaulding *et al*., 2009) together with 10 orders of land mammals, 6 of which are ruminants (Tab. 1).

## Ascent of the Bovidae

**Figure 1:**
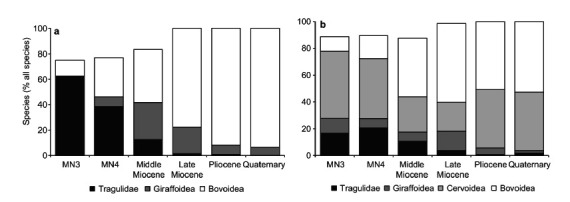
Relative abundance of tragulids, giraffids, cervids and bovids in different geological periods (expressed as a percentage of all ruminants) in Africa (panel a) and Eurasia (panel b). MN3 and MN4 represent zones in the Early Miocene. The difference to 100% comprises pecoran species with unknown taxonomic affiliation, species from extinct pecoran families and a negligible number of musk deer (Moschidae). Reproduced with permission from (Clauss and Roessner, 2014) © Finnish Zoological and Botanical Publishing Board 2014.

Despite recent species splitting (Groves and Grubb, 2011; Burgin, *et al*., 2018), the number of genera of terrestrial artiodactyls has remained rather constant (Tab. 1). The five families with diffuse placentation have few living representatives. In contrast, more than half the genera and 65% of recognized species are from Family Bovidae. Bovids have not always been so prevalent and it is instructive to compare the ruminant fauna of earlier epochs (Clauss and Roessner, 2014) (Fig. 1). In Africa during the Early Miocene (MN3 and MN4 in Fig. 1a) there was a preponderance of tragulids (present-day chevrotains). Tragulids are basal to pecoran ruminants and have a diffuse placenta without placentomes. Their relative abundance was greatly reduced by the Middle Miocene when there were roughly equal numbers of giraffids and bovids. The only giraffids living today are the okapi and four species of giraffe, but giraffids were more abundant and diverse in earlier epochs. What explains their fate? One suggestion is that a longer gestation period put them at a disadvantage compared to bovids, which could better adapt to climate change by adopting seasonal breeding patterns (Clauss and Roessner, 2014). This might also explain why giraffids were never numerous in the Eurasian fauna (Fig. 1b), where they faced additional competition from deer and their kin (Cervidae). 

**Table 1:** Terrestrial mammals of Order Cetartiodactyla (Burgin *et al*., 2018). In addition, the order includes 11 families (40 genera) of whales and dolphins with diffuse placentation.

Family	Common names	Genera	Species	Placentation
Camelidae	Camels, llamas	2	7	Diffuse
Tayassuidae	Peccaries	3	5	Diffuse
Suidae	Swine	6	28	Diffuse
Hippopotamidae	Hippopotamuses	2	4	Diffuse
Tragulidae	Chevrotains	3	10	Diffuse
Moschidae	Musk deer	1	7	Oligocotyledonary
Giraffidae	Giraffes, okapi	2	5	Polycotyledonary
Antilocapridae	Pronghorn	1	1	Polycotyledonary
Cervidae	Deer	18	93	Oligocotyledonary
Bovidae	Antelopes, cattle, sheep and goats	54	297	Polycotyledonary

## Classification of Bovidae

**Figure 2:**
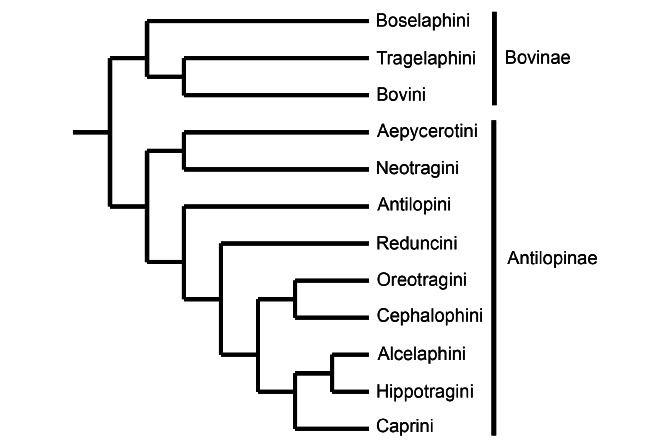
Phylogenetic tree for the two subfamilies and 12 tribes of family Bovidae. The branch order is derived from Hassanin *et al*. (Hassanin *et al*., 2012). Branch lengths are arbitrary and do not refer to a time scale.

There are 12 clades of bovids (Fig. 2). They sometimes are afforded the status of subfamilies (Wilson and Reeder, 2005), but the current consensus has them as tribes grouped in two subfamilies (Groves and Grubb, 2011; Hassanin *et al*., 2012; Bibi, 2013). Bovinae is reserved for cattle (Bovini), spiral-horned, antelope-like bovines (Tragelaphini) and the nilgai (*Boselaphus tragocamelus*) and four-horned antelope (*Tetracerus quadricornis*) (Boselaphini). Antilopinae encompasses 9 tribes of antelopes including sheep and goats. Molecular phylogenetics affords no justification for separate treatment of sheep and goats (Caprini), which can be characterized as, “medium-sized, thick-legged antelopes with limbs and hooves modified for climbing and leaping over rough or stony ground” (Kingdon, 1997).

## Domestication

Why were some ruminants domesticated and others not? Most domesticated animals (Tab. 2) are derived from species that are highly social and live in herds with a dominance hierarchy (cats are a notable exception). Another factor is flight distance: it is shorter in goats, which rely on agility to escape from predators, than in gazelles, which rely on speed. This could explain why goats were tamed and gazelles were not, although both had been a major food source for hunter-gatherers (Clutton-Brock, 2012). The transition from a hunter-gatherer economy to herding was regional and may have been precipitated by overhunting of the wild mammals. It occurred at several places from the Near East through South and Southeast Asia (Clutton-Brock, 2012). However, domestication did not occur south of the Sahara, where bovids make up a large part of the fauna. 

A trait shared by domesticates of many species is the retention of juvenile traits in the adult (neotony). This extends to behaviour as much as to anatomical features. It likely results from selection for tameness and against aggressive behaviour (Budiansky, 1994). The most convincing evidence comes from an experiment on the silver fox (*Vulpes vulpes*) where selection for tameness over many generations resulted in foxes that behaved much like domestic dogs (Belyaev, 1979). The experiment was recently revisited with a view to identifying genes associated with tame and aggressive behaviours (Kukekova *et al*., 2018). 

The first bovids to be domesticated were goats and sheep. Some of the earliest evidence for both is from Jericho (Clutton-Brock, 2012). However, genomic evidence points to three centres of goat domestication within the Fertile Crescent (Daly *et al*., 2018). Domestication of taurine cattle occurred in the Near East and of humped or zebu cattle in present day Pakistan. They are thought to have been derived from two species of aurochs (*Bos primigenius* and *B. namadicus*) (Clutton-Brock, 2012; Pitt *et al*., 2018). Cattle were introduced to other regions as herding spread to Europe and North Africa and throughout Asia. Thus, taurine and zebu cattle were introduced independently to southern Africa via the Sahara and the Horn of Africa and at least 120 breeds developed there. A separate domestication of taurine cattle in Egypt is not supported by current analyses of genetic data (Pitt *et al*., 2018). Elsewhere there was domestication of the yak in Tibet, Bali cattle in Indonesia and of water buffalo both in China (the swamp buffalo) and South Asia (the river buffalo). The gayal of South and Southeast Asia is a semi-domesticated gaur (Tab. 2). 

There has, of course, been a great deal of cross-breeding and not just between taurine and zebu cattle. As an example, introgression has occurred between yak and Tibetan cattle (Wu *et al*., 2018). The latter have acquired haplotypes of two genes in the hypoxia-inducible factor pathway: *EGLN2* and *HIF3a*. One consequence of this is a reduced haematopoietic response to hypoxia, which confers an advantage to cattle living at high altitude. Introgression has also occurred between cattle and wild species, including the European bison or wisent (Wu *et al*., 2018).

Livestock biomass, 80% of it from cattle, buffaloes, sheep and goats, greatly exceeds the biomass of all wild mammals (0.1 versus 0.007 gigatons of carbon) (Bar-On *et al*., 2018). Budiansky has argued, “domestication seems natural only because it happened, but it happened only because it was natural” (Budiansky, 1994). To the extent that animals were complicit in the domestication process, as he contends, this was a highly successful strategy.

**Table 2:** Domestication of bovids (Clutton-Brock, 2012).

Common Name	Latin Name	Species from which Derived	Latin Name	Where Domesticated
Taurine cattle	*Bos taurus*	Aurochs	*Bos primigenius*	Near East and possibly North Africa
Zebu cattle	*Bos indicus*	Indian Aurochs	*Bos namadicus*	South Asia (Pakistan)
Domestic Yak	*Bos grunniens*	Wild Yak	*Bos mutus*	Tibet and Nepal
Gayal	*Bos frontalis*	Gaur	*Bos gaurus*	South and Southeast Asia (semi-domesticated)
Bali Cattle	*Bos javanicus*	Banteng	*Bos javanicus*	Indonesia
Water Buffalo	*Bubalus bubalis*	Asian Wild Water Buffalo	*Bubalus arnee*	China or Southeast Asia (Swamp Buffalo); South Asia (River Buffalo)
Domestic Goat	*Capra hircus*	Bezoar Goat	*Capra aegagrus*	Near East
Domestic Sheep	*Ovis aries*	Asiatic Mouflon	*Ovis orientalis*	Near East

## Placentation

Most domesticated bovids have a bicornuate uterus with the placenta extending from the pregnant to the non-pregnant horn. There are four rows of caruncles in each horn and placentomes are 80-110 in number. This pattern was likely present in the common ancestor of pecoran ruminants (Klisch and Mess, 2007).

Large, grazing antelopes of the Tribe Hippotragini, such as the sable antelope (*Hippotragus niger*) and some members of Alcelaphini, such as black wildebeest (*Connochaetes gnou*), have a uterus duplex with a bifurcated cervical canal (Hradecky, 1982). This restricts the placenta to the gravid horn (Hradecky, 1983a). Interestingly, the fetal membranes are also restricted to one horn in the hartebeest (*Alcelaphus buselaphus*), an antelope with a bicornuate uterus (Hradecky, 1983a). In Hippotragini, perhaps as a compensation, there are 6-8 rows of caruncles and the placenta has a larger than average number of placentomes (Hradecky, 1983a, 1983b). 

In contrast, antelopes of the Tribe Reduncini, such as the kob (*Kobus kob*) and waterbuck (*K. ellipsirymnus*), have just two rows of caruncles in each horn with the placenta comprising 10-20 rather large placentomes (Hradecky, 1983a, 1983b).

## Unilateral implantation

Kobs and waterbucks have bicornuate uteri and ovulation alternates between right and left ovaries, yet implantation invariably occurs in the right horn (Buechner, 1961; Spinage, 1969). A similar pattern of bilateral ovulation with right-sided implantation occurs in several tribes of antelope including the impala (*Aepyceros melampus*) (Mossman and Mossman, 1962), suni (*Neotragus moschatus*) (Loskutoff *et al*., 1990), Kirk’s dikdik (*Madoqua kirkii*) (Kellas, 1955), and bush duiker (*Sylvicapra grimmia*) (Child and Mossman, 1965; Symington and Paterson, 1970) (Tab. 3). In the suni, the left horn is smaller than the right and its caruncles less well developed (Loskutoff *et al*., 1990). Similarly, in non-pregnant impala, the caruncular epithelium is thinner in the left horn than the right (Jones *et al*., personal communication). Unilateral implantation in a bicornuate uterus is not confined to bovids. It is also known to occur in camelids (Picha *et al*., 2013), toothed whales (Ohsumi, 1964) and bats (Rasweiler and Badwaik, 2000).

## Litter size

The reproductive strategy of ruminants entails a long gestation with a singleton fetus that is precocial at birth. This pattern was present in the common ancestor of Ferungulata and only pigs have reverted to a larger litter of three or more offspring (Klisch and Mess, 2007; Carter and Mess, 2017). Among the Bovidae, singleton pregnancy is the rule. Exceptions include twinning in the nilgai and four-horned antelope (Boselaphini) as well as some gazelles (Antilopini). Domestic sheep and goats (Caprini) bear twins and sometimes triplets, but many wild caprines have singleton pregnancies (Castello, 2016). In cattle and rarely in other ruminants, pigs and camels, vascular connections between twins of separate sex result in masculinization of the female reproductive tract. Freemartins are not known from other orders of mammal (Padula, 2005).

## Binucleate trophoblast cells

Binucleate trophoblast cells (BNCs) were first described for the sheep placenta by Assheton (Assheton, 1906). Their critical role in ruminant placentation was not fully understood until the work of Wooding in sheep (Wooding, *et al*., 1980) and cattle (Wooding and Wathes, 1980; Wooding, 1982). This established that BNCs migrate towards and fuse with uterine epithelial cells to form a fetomaternal hybrid - either a syncytium or trinucleate cells. Some authors favour referring to BNCs as trophoblast giant cells (Klisch *et al*., 1999). 

Because the maternal-fetal interface in ruminants contains hybrid elements, some authors recommend the term “synepitheliochorial” (Wooding and Burton, 2008), reserving epitheliochorial for species where only trophoblast and maternal epithelium occur at the interface (Carter and Enders, 2013). 

BNCs occur in all ruminants, including chevrotains, where they contribute to a fetomaternal syncytium (Kimura *et al*., 2004; Wooding *et al*., 2007). A syncytium forms at early stages of implantation in deer and cattle and in sheep and goats it persists until later in gestation (Wooding *et al*., 1980; Wooding, 1982), whereas trinucleate cells are found in cattle (Wooding and Wathes, 1980) and deer (Wooding *et al*., 2018). Although this is the usual outcome in cattle, multinucleate cells can be observed that seemingly result from fusion of additional BNCs with trinucleate cells (Klisch *et al*., 1999). The trinucleate cells of cattle and deer atrophy and die after releasing their granules and are reabsorbed by the trophectoderm ( Wooding and Wathes, 1980; Wooding, 1982).

**Figure 3:**
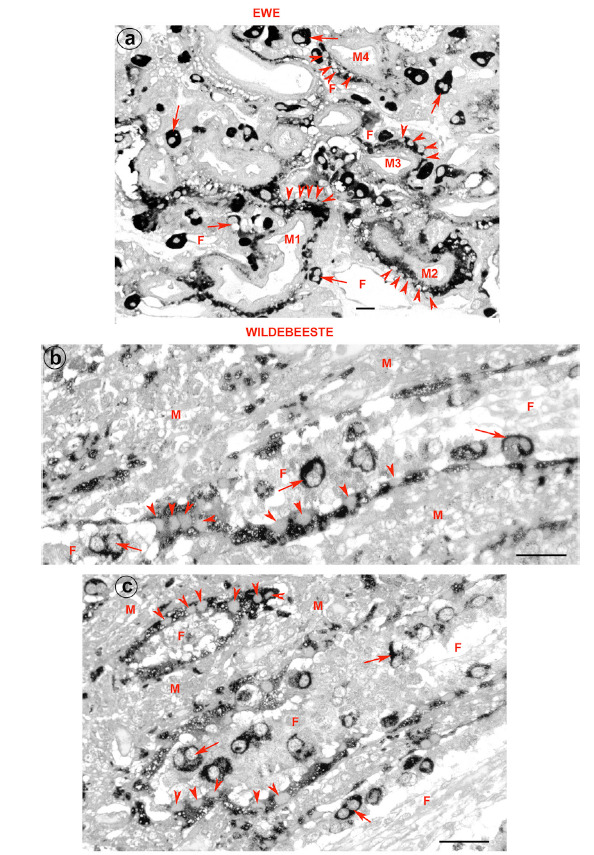
Sections through the placentomes from sheep (*Ovis aries*) and blue wildebeest (*Connochaetes taurinus*) immunostained with a PAG antibody. (a) Sheep: the fetomaternal syncytium between trophoblast and uterine stroma is continuously stained by PAG and can best be seen around the four maternal blood vessels M1, M2, M3 and M4. The nuclei in the syncytium (arrowheads) are fairly evenly spaced. F marks fetal blood vessels; Arrows, BNC. Bar = 30 µm. (b) and (c) Blue wildebeest: as in the ewe the fetomaternal syncytium is continuously stained for PAG and separates the maternal (M) and fetal (F) tissues. Nuclei (arrowheads) are scattered randomly along the syncytium. Arrows mark the BNC. Bar = 60 µm. Reproduced from (Wooding *et al*., 2018) © 2018 with permission from Elsevier.

It has been difficult to discuss the alternative fates of BNCs in an evolutionary context, since comparative studies of bovid placenta tend to document the presence of binucleate cells, but not their subsequent fate (Hradecky, 1986; Hradecky *et al*., 1988b; Benirschke, 2012). Recently, a survey was made of a wide range of ruminants including a chevrotain (Tragulidae), eight bovids (Bovidae), eight deer (Cervidae), the pronghorn (Antilocapridae) and a giraffe (Giraffidae) (Wooding *et al*., 2018). This study used antibodies raised against pregnancy-associated glycoproteins and immunogold staining to localise granules in BNCs, trinucleate cells and syncytium. A fetomaternal syncytium was confirmed for the chevrotain, but most of the pecoran ruminants had trinucleate cells. Exceptions were the sheep and the blue wildebeest (*Connochaetes taurinus*) (Fig. 3). 

The BNCs of ruminants are an evolutionary novelty. They do not occur in other even-toed ungulates nor are they related to the multinucleate giant cells of camelids (Klisch and Mess, 2007). Since they migrate to and fuse with the uterine epithelium, the population of BNCs requires continual renewal. In their early developmental stages, BNCs of cattle retain contact with the basal membrane of the trophoblast (Attiger *et al*., 2018). This raises the possibility that BNCs arise by mitosis of basally located stem cells rather than being derived from uninucleate trophoblast cells (Attiger *et al*., 2018).

**Table 3:** Variations in placentation across the bovid family. Hradecký is the source for data on villous structure (Hradecky *et al*., 1988b).

Tribe	Description	Gross anatomy	Villous structure
Subfamily Bovinae			
Boselaphini	Nilgai, four-horned antelope	Twins; low number of placentomes in four-horned antelope	
Tragelaphini	Spiral-horned, antelope-like bovines: common eland, greater kudu, nyala, sitatunga		Long villi (10 mm) (eland, bongo, nyala) to moderate villi (7 mm) (greater kudu) with moderate branching in both
Bovini	Bison and cattle, buffaloes, saola		Long villi (up to 17 mm) with extensive branching (domestic cattle)
Subfamily Antilopinae			
Aepycerotini	Single species: impala	Implantation in the right horn	Long villi (10 mm) with intricate branching
Neotragini	Forest-dwelling dwarf antelopes: suni, royal antelope	Implantation in the right horn in suni; few placentomes in royal antelope	
Antilopini	Large tribe with 13 genera: gazelles, dik-diks and saiga	Twinning occurs in some gazelles, steenboks and saigas; implantation in the right horn in Kirk’s dik-dik	Short villi (2.5 mm) with extensive branching (steenbok)
Reduncini	Kobs, reedbucks, rhebok	Two rows of caruncles; 10-20 placentomes; implantation in the right horn	Long villi (15 mm) with almost no branching (kob, lechwe, Bohor reedbuck, waterbuck,)
Oreotragini	Single species: klipspringer	Few cotyledons	
Cephalophini	Forest-dwelling duikers	Implantation in the right horn and few placentomes in bush duiker	Long villi (10 mm) with little branching (bush, bay and Maxwell’s duikers)
Alcelaphini	Medium to large sized antelopes: wildebeest, hartebeest	Uterus duplex in wildebeests; unilateral implantation	Medium villi (8 mm) with moderate branching (black wildebeest)
Hippotragini	Large grazing antelopes: sable and roan antelopes	Uterus duplex and unilateral placentation; 6-8 rows of caruncles; 109-185 placentomes	Short villi (4 mm) with numerous, long branches (sable antelope)
Caprini	Large tribe with 12-14 genera: sheep, goats and muskoxen	Twinning common in many species including domestic sheep and goat	Numerous fine branches (domestic sheep)

## Syncytins

Fusion of cells from two individuals to form a viable unit seems problematic. The explanation seems to lie in expression by the binucleate cell of one or more syncytins. These are coded by genes of retroviral origin that have been incorporated into the genome and are expressed in the placenta where they promote cell fusion. Syncytin genes occur in primates, rodents, rabbits, carnivores and even in a marsupial and a lizard (Dupressoir *et al*., 2012; Cornelis *et al*., 2015; Cornelis *et al*., 2017). They are not orthologous genes as each represents an independent capture from a retrovirus (Lavialle *et al*., 2013). Syncytins are encoded by retroviral *env* genes. In a retrovirus, the envelope protein is responsible for fusion with a host cell (Dupressoir *et al*., 2012). Most syncytins function to promote fusion of trophoblast cells to form multinucleate syncytiotrophoblast. In pecoran ruminants, however, a syncytin (*Syncytin-Rum1*) was found that enables the binucleate cell to fuse with a uterine epithelial cell. The envelope protein has an immunosuppressive domain, and this is highly conserved in ruminant syncytin (Cornelis *et al*., 2013).

A second syncytin gene (*Fematrin-1*) is expressed by BNCs in taurine cattle, Bali cattle and water buffalo (Bovini) and sitatunga (*Tragelaphus spekii*) (Tragelaphini), although not in domestic sheep and goat (Caprini) (Nakaya *et al*., 2013). It was suggested that this gene could account for the formation of trinucleate cells in bovines as opposed to syncytial plaques in caprines (Nakaya *et al*., 2013). However, that interpretation is not compatible with recent work (Wooding *et al*., 2018), from which it is clear that trinucleate cells are basal and syncytium formation is the derived state. 

It is not possible to pinpoint the time when a retrovirus became incorporated in the genome. A provisional estimate for *Syncytin-Rum1* is more than 30 million years ago (mya) (Cornelis *et al*., 2013) and for *Fematrin-1* 18.3-25.4 mya (Nakaya *et al*., 2013).

## Variations in placentation across the bovid family

There are 3 tribes in Subfamily Bovinae and 9 tribes in Antilopinae (Fig. 2; Tab. 3). In contrast to the substantial literature on domestic cattle and sheep (Wooding and Burton, 2008), relatively few sources exist for antelope-like bovines and antelopes. In Subfamily Bovinae there are reasonable accounts of placentation in domestic yak (Liu *et al*., 2010), water buffalo (Carvalho *et al*., 2006; Pereira *et al*., 2010) and African buffalo (*Syncerus caffer*) (Schmidt *et al*., 2006). Subfamily Antilopinae is poorly covered with detailed accounts only for impala (Kayanja and Epelu-Opio, 1976), dik-diks (*Madoqua* spp.) (Sedlaczek, 1912; Wislocki, 1941; Kellas, 1955), oribi (*Ourebia ourebi*) (Kellas, 1966), Bohor reedbuck (*Redunca redunca*) (Sedlaczek, 1912) and roan antelope (*Hippotragus equinus*) (Sedlaczek, 1912). The first comprehensive account of placentation in ruminants was that of Andresen (Andresen, 1927). However, the broadest approach so far is that of Hradecký (Hradecky, 1986; Hradecky *et al*., 1988a, 1988b) and much of the information below has been gleaned from his thesis (Hradecky, 1986) and from re-examination of his slides in the Harland W. Mossman Collection at the University of Wisconsin Zoological Museum. Another important source is the web site established by the late Kurt Benirschke (Benirschke, 2012); it covers many bovids, but is based largely on examination of the fetal membranes at delivery. 

## Placentome number

Placentome number varies considerably within and between species, but lies mainly in the range 50-175 placentomes. Several antelopes, however, approach an oligocotyledonary state. Thus 6 placentomes were reported in a bush duiker (Cephalophini) (Starck, 1959), 6-8 in the Royal antelope (*Neotragus pygmaeus*) (Neotragini) (Benirschke, 2012), 14 in the klipspringer (*O. oreotragus*) (Oreotragini) and 28 in Phillips’s dik-dik (*Madoqua phillipsi*) (Antilopini) (Starck, 1959). As mentioned above, a low cotyledon number is usual in the Reduncini. In addition, an early publication remarked on the moderate number of placentomes in the four-horned antelope (Boselaphini) and recognized the importance of the interplacentomal regions of the allantochorion (Weldon, 1884). 

## Placentome shape

There are three basic shapes to placentomes (Andresen, 1927; Mossman, 1987). The convex type is typical of cattle and often has a narrow base giving it a mushroom shape. Flat placentomes are found mostly in deer, and the concave type is found in sheep and goats. The sheep placentome has a central concavity where extravasated maternal erythrocytes are taken up and processed by columnar trophoblast cells (Burton *et al*., 1976; Myagkaya and Vreeling-Sindelarova, 1976). Many antelopes have concave placentomes, examples being Kirk’s dik-dik (Antilopini), the roan antelope (Hippotragini) and the topi (*Damaliscus lunatus*) (Alcelaphini) (Andresen, 1927). It is not known if these placentomes have a central haemophagous region like that of the sheep.

## Villus complexity

**Figure 4:**
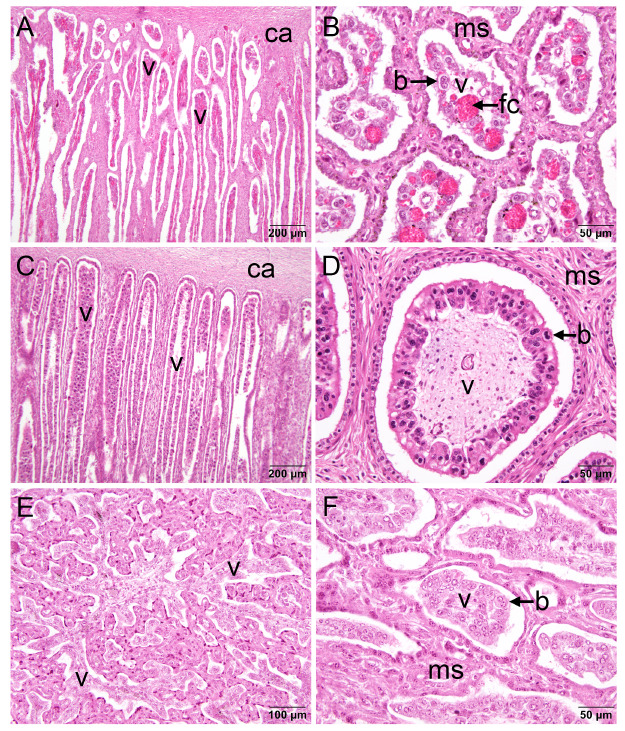
Histology of the placentome in three bovids; 6 µm sections stained with haematoxylin and eosin. A. Longitudinal section of a placentome from the common eland (*Taurotragus oryx*) in late gestation (M26615; eland 75 in (Hradecky *et al*., 1988b)). Note the branching of the villi that is typical of Subfamily Bovinae. B. Cross section of a placentome from the same animal. C. Longitudinal section of a placentome from the kob (*Kobus kob*) at 97 days gestation; fetal length 23.4 cm (M26812; kob 7 in (Hradecky *et al*., 1988b)). Note the straight villi with minimal branching known only from impala and duikers. D. Cross section of a placentome from the kob at 127 days gestation; fetal length 34.5 cm (M26814; kob 9 in (Hradecky *et al*., 1988b)). E. Longitudinal section of a placentome from the impala (*Aepyceros melampus*) in mid-gestation; fetal length 11 cm (M26463; impala 118 in (Hradecky *et al*., 1988b)). Extensive branching of the villi is representative of the pattern in most antelopes. F. Cross section of a placentome from the same animal. Labels: b, binucleate trophoblast cell; ca, base of caruncle; fc, fetal capillary; ms, maternal stroma; v, villus. Hradecký voucher specimens in the Harland W. Mossman Embryological Collection, University of Wisconsin Zoological Museum.

Hradecký paid close attention to the branching patterns of the villi (Hradecky, 1986; Hradecky *et al*., 1988b). In domestic cattle, villi were up to 17 mm long with complex branching. In two antelope-like bovines, common eland (*Taurotragus oryx*) and greater kudu (*Tragelaphus strepsiceros*), villi were 7-10 mm long and moderately branched (Fig. 4 A-B). A similar pattern was found in bongo (*T. eurycerus*), nyala (*T. angassii*) and sitatunga. 

Branching patterns were much more varied among the antelopes. An interesting example was Reduncini where only 10-20 placentomes were formed. One might anticipate a complex internal structure to maximize the maternal-fetal interface, but the reverse was the case. In the kob, villi were up to 15 mm long and coursed through the entire depth of the placentome with minimal branching (Fig. 4C). Villi were evenly distributed and averaged 200 µm in diameter at half height (Fig. 4D). The same applied to the lechwe (*Kobus leche*), waterbuck and Bohor reedbuck. A similar pattern was found in the unrelated bush duiker (Cephalophini), where the villi were up to 10 mm long with only slight branching and 200-300 µm in diameter at half height. Findings were similar in bay duiker (*Cephalophus dorsalis*) and Maxwell’s duiker (*Philantomba maxwellii*).

In contrast, the villi were extensively branched in the impala (Aepycerotini), where the villi were up to 10 mm long (Fig. 4 E-F) and in the steenbok (*Raphicerus campestris*) (Antilopini), where they were up to 2.5 mm long. Moderate branching occurred in the black wildebeest (Alcelaphini) and sable antelope (Hippotragini) with villi 8 and 4 mm in length, respectively. 

## Placental hormones

Placental hormones act to adapt maternal physiology to pregnancy and lactation (Napso *et al*., 2018). In bovids, interferon-tau from the trophectoderm of the elongated blastocyst acts as a luteotrophic factor (Roberts *et al*., 2003). Peptide hormones secreted by the placenta proper include pregnancy-associated glycoproteins (PAGs), placental lactogens, prolactin-like proteins, and placental growth hormone. The coding genes evolved from non-placental ones through one or more rounds of gene duplication.

## Placental lactogens

Placental lactogens have arisen by convergent evolution in primates, rodents and ruminants (Soares, 2004). In ruminants, tandem duplication of the prolactin gene has given rise to genes for placental lactogen (PL) and a family of prolactin-like proteins.

A survey of placental lactogens in mammals was made by Forsyth (Forsyth, 1986). She found PL to be present in 11 species of bovids including cattle and African buffalo (Bovini), Thomson’s gazelle (*Eudorcas thomsonii*) (Antilopini), blue wildebeest (*Connochaetes taurinus*) (Alcelaphini), the roan antelope (Hippotragini), several sheep and goats and the muskox (*Ovibos moschatus*) (Caprini). The sequences of bovine and ovine PLs are very different, suggesting a high rate of molecular evolution (Wallis, 1993). Moreover, bovine PL is heavily glycosylated whereas ovine PL is not (Colosi *et al*., 1989). Within the placentome, ovine PL is located exclusively to the BNCs and the syncytium formed by fusion of BNCs with uterine epithelial cells (Wooding *et al*., 1992). Bovine PL is similarly restricted to BNCs and the hybrid trinucleate cells (Wooding and Beckers, 1987). Indeed, fusion of BNCs with maternal epithelium has been proposed as a mechanism for delivering PL and other placental hormones to the maternal tissues (Wooding and Beckers, 1987).

The best documented role of ovine prolactin is to stimulate the secretion of histotrophe (“uterine milk”) by binding to prolactin receptors in the uterine glands (Noel *et al*., 2003). PL can be detected in the maternal plasma of sheep, but seems to have little effect on mammary gland development (Min *et al*., 1997). Bovine PL is barely detectable in maternal plasma (Byatt *et al*., 1987) so may act only in a paracrine manner. In contrast, the convergently evolved PLs of rodents are important for pregnancy maintenance (Galosy and Talamantes, 1995) and mammary gland development (Thordarson *et al*., 1989). Human and primate PLs (evolved by duplication of the growth hormone gene) act on maternal metabolism to improve nutrient availability to the fetus (Handwerger, 1991). 

Ovine and bovine PLs do reach the fetal circulation, although by an unknown mechanism, and may promote fetal growth (Taylor *et al*., 1980; Byatt *et al*., 1987).

## Placental prolactin-like proteins

Unlike PLs, the prolactin-like proteins (PRPs) do not bind to prolactin or growth hormone receptors and their function is unclear. No less than 12 PRP genes are expressed in bovine placenta (Ushizawa *et al*., 2005; Larson *et al*., 2006). Sheep and goat have two PRP genes each and they are homologous with genes in cattle (Ushizawa *et al*., 2007a; Ushizawa, *et al*., 2007b). Expression of the PRPs is limited to BNCs (Milosavljevic *et al*., 1989; Ushizawa *et al*., 2007a; Ushizawa *et al*., 2007b).

## Placental growth hormone

A placental growth hormone has been described in domestic sheep and goat. The protein was localized to uninucleate and binucleate trophoblast cells and the syncytium of the ovine placentome (Lacroix *et al*., 1996). It is possible that gene duplication took place during the evolution of caprine ruminants (Wallis *et al*., 1998), but it could have a deeper origin as discussed below. Uterine gland hyperplasia and histotrophe secretion in sheep require the sequential action of interferon-tau, ovine PL and ovine placental growth hormone (Noel *et al*., 2003).

## Pregnancy-associated glycoproteins

Pregnancy-associated glycoproteins (PAGs) are placental hormones that evolved in the common ancestor of Cetartiodactyla by duplication of the pepsin-F gene, which codes for an aspartic proteinase (Hughes *et al*., 2003; Wallace *et al*., 2015). There were two further rounds of gene duplication. The first gave rise to the “ancient PAGs,” which retained the active site of the proteinase. The second occurred in the ruminant lineage and many of these “modern PAGs” lack the active site (Wallace *et al*., 2015). Bovids have a high number of PAG genes: 18 protein-coding genes and 14 pseudogenes have been identified in taurine cattle (Telugu *et al*., 2009). Multiple genes were revealed by Southern blotting of genomic DNA from a wide selection of bovids (Tab. 4) (Xie *et al*., 1997), but detailed studies have been confined to domestic species including sheep and goats (Xie *et al*., 1997; Tandiya *et al*., 2013). Antibodies raised against bovine PAG-1 and ovine PAG-2 have been used for immunolocalization studies across a range of species (Tab. 4; Wooding *et al*., 2018), but the full range of PAG proteins remains to be explored. 

Nevertheless, there is an interesting distribution of gene expression between trophoblast of the cotyledons and intercotyledonary areas ( Wooding *et al*., 2005; Touzard *et al*., 2013). In bovine placenta, modern PAGs were transcribed mainly in the cotyledons and ancient PAGs in the intercotyledonary areas; the exception was ancient PAG-2, which was expressed in the cotyledons (Touzard *et al*., 2013). The cellular localization of representative PAG proteins also differed. Thus PAG-1 (ancient) was detected in the cytoplasm of BNCs from the intercotyledonary chorion. PAG-11 (modern) was detected in BNCs from both cotyledonary and intercotyledonary BNCs; however, no BNC stained for both PAG-1 and PAG-11. Finally, PAG-2 (ancient) was found in the uninucleate trophoblast cells of the cotyledons, but appeared to be excluded from BNCs (Touzard *et al*., 2013).

The function of PAGs remains unclear. In cattle, two of the ancient PAGs are more highly expressed in cotyledons from early gestation, perhaps implying a role in placentation (Wiedemann *et al*., 2018). Further, it has been suggested that ancient PAGs act as linking molecules at the fetomaternal interface and that modern PAGs have an immunomodulatory function (Wooding *et al*., 2005). These proteins are produced in large quantities and some are secreted to the maternal circulation, where they can be used to monitor pregnancy (Wallace *et al*., 2015).

**Table 4:** Pregnancy-associated glycoproteins in bovids (Xie *et al*., 1997; Wooding *et al*., 2005; Wooding *et al*., 2018)

Tribe	Multiple PAGS demonstrated by Southern blotting	PAGs localized by immunohistochemistry
Subfamily Bovinae		
Boselaphini	No data	Nilgai
Tragelaphini	Nyala	No data
Bovini	Taurine cattle, gaur, yak, African buffalo	Domestic cattle, African buffalo, American bison
Subfamily Antilopinae		
Aepycerotini	Impala	Impala
Neotragini	No data	No data
Antilopini	Springbok	Springbok
Reduncini	No data	No data
Oreotragini	No data	No data
Cephalophini	A duiker	No data
Alcelaphini	Black wildebeest	Blue wildebeest
Hippotragini	No data	No data
Caprini	Domestic sheep, Dall’s sheep, Markhor goat, goral, takin	Domestic sheep

## Conclusions

Many key features of placentation had evolved before Bovidae emerged as a separate family (Carter, 2014; Carter and Mess, 2017; Tab. 5). Even so, there are several interesting innovations in one or more of the twelve tribes. One can estimate when they emerged by reference to the current tree (Fig. 2), which follows the phylogeny of Hassinin *et al*. (Hassanin *et al*., 2012). There are some differences in branching order in the tree offered by Bibi (Bibi, 2013), such as a more basal position for Caprini, but these do not affect the arguments in this section.

One curious example is the appearance in Hippotragini and some members of Alcelaphini of a uterus duplex with a bifurcated cervical canal (Hradecky, 1982), restricting the placenta to the gravid horn (Hradecky, 1983a). This feature could have emerged in the common ancestor of the two tribes as even the hartebeest, with its bicornuate uterus, has the fetal membranes restricted to one horn (Hradecky, 1983a). 

In many other species of Suborder Antilopinae, implantation usually occurs in the right horn, although ovulation alternates between right and left ovaries and the fetal membranes can extend to the left horn. Exceptions are not well documented. In the oribi, for example, implantation was clearly in the right horn of three females whereas a fourth carried twins, one confined to the right horn while the other fetus “occupied a portion of both horns” (Kellas, 1966). A proclivity to right-sided implantation may therefore have arisen after the common ancestor of antelopes diverged from that of bovines with reversal occurring mainly in species, such as domestic sheep, where twinning frequently occurs. 

Another significant feature concerns the fate of BNCs. As recently documented (Wooding *et al*., 2018), the usual pattern is fusion with a uterine epithelial cell to form a trinucleate cell. In sheep and goats, however, a fetomaternal syncytium is formed that persists throughout gestation. The same happens in the blue wildebeest (Wooding *et al*., 2018). It is, therefore, notable that these species represent two tribes (Caprini and Alcelaphini) that share a common ancestor with a third (Hippotragini) (Fig. 2; Hassanin *et al*., 2012; Bibi, 2013). It seems likely that the most recent common ancestor of these three tribes had a persistent fetomaternal syncytium.

Most placental hormones predate the emergence of Bovidae (Tab. 5). An interesting exception is placental growth hormone. As noted above, it is likely that duplication of the growth hormone gene took place during the evolution of caprine ruminants (Wallis *et al*., 1998). However, as there is no data for antelopes, it could have a deeper origin. Therefore, it would be especially interesting to know if placental growth hormone is present in the wildebeest; as noted it shares a common ancestor with caprines (Hassanin *et al*., 2012) and like them forms a fetomaternal syncytium (Wooding *et al*., 2018). The sequences of placental lactogen and degree of glycosylation differ between domestic cattle and sheep, but there is insufficient data to show when the divergence occurred and whether it might be linked to the emergence of a placental growth hormone in the latter.

Fewer evolutionary novelties have been observed in Subfamily Bovinae. The capture of an *env* gene with the properties of a syncytin (*Fematrin-1*) is the most convincing example, as it is present in taurine cattle, Bali cattle and water buffalo (Bovini) and also in the sitatunga (Tragelaphini) (Nakaya *et al*., 2013). There are more PAGs in cattle than in sheep, but it is not clear when the tandem duplication of PAG genes took place.

As discussed elsewhere (Carter and Enders, 2013), it is difficult to identify the selection pressures behind placental evolution. One suggestion has been that evolution in cotyledon number, shape and interdigitation of fetal villi and maternal crypts has been driven by maternal-fetal competition for nutrients (Klisch and Mess, 2007). The assumption is that allocation of resources to the fetus will increase the fitness of the offspring, but decrease the mother’s fitness for future reproduction (Haig, 2008). The convergent evolution of placental lactogens and growth hormones in rodents, ruminants and primates can also be interpreted as attempts by the fetus to influence maternal acquisition and allocation of nutrients (Haig, 2008; Napso *et al*., 2018). Elsewhere it was argued that the interplay between the fetal semi-allograft and the maternal immune system is a significant force in placental evolution (Carter and Enders, 2013). An advantage of epitheliochorial over more invasive placentation is that it allows the immune system to be primed against uterine infection rather than suppressed to allow trophoblast invasion (Carter and Enders, 2013). Fusion of BNCs with uterine epithelium exposes trophoblast to the immune system, but the process is aided by syncytins with immunomodulatory properties (Cornelis *et al*., 2013) and the modern PAGs may also act in this fashion (Wooding *et al*., 2005). Unfortunately, little is known about the immune system of the uterus even in cattle and sheep. For the antelopes and antelope-like bovines that live in the wild this is one of many aspects that deserves further study. 

**Table 5:** Timeline of placental evolution, showing the appearance of distinct characters in various taxonomic clades (Carter, 2014). Approximate dates (million years ago, mya) above the family level are taken from Meredith *et al*. (Meredith *et al*., 2011) and for families and subfamilies from Bibi (Bibi, 2013).

Taxonomic clade	Branching point (mya)	Geological period	Character
Fereuungulata^1^	82.0	Cretaceous	Epitheliochorial placentation
Cetartiodactyla	65.4	Palaeocene	Pregnancy-associated glycoproteins
Ruminantia	40.3	Eocene	Binucleate trophoblast cells and fusion with uterine epithelium
			Placental lactogens
Pecora	20.4	Early Miocene	Placentomes
			Syncytin gene (*Syncytin-Rum1*)
			Interferon-tau gene (*IFNT*)
Bovidae	16.2	Early Miocene	Duplication of HBB gene for high affinity fetal hemoglobin
Bovinae	11.0	Middle Miocene	Syncytin gene (*Fematrin-1*)
Caprini	10.1	Middle Miocene	Placental growth hormone

1 Fereuungulata comprises the orders Perissodactyla, Pholidota, Carnivora and Cetartiodactyla.
